# Analysis of the effectiveness of ultrasound and clinical examination methods in fetal weight estimation for term pregnancies

**DOI:** 10.4274/tjod.28044

**Published:** 2015-12-15

**Authors:** Mehmet Zahran, Yusuf Aytaç Tohma, Salim Erkaya, Özlem Evliyaoğlu, Eser Çolak, Bora Çoşkun

**Affiliations:** 1 Etlik Zübeyde Hanım Womens’s Health Teaching and Research Hospital, Department of Obstetrics and Gynecology, Ankara, Turkey; 2 Başkent University Faculty of Medicine, Department of Obstetrics and Gynecology, Konya, Turkey

**Keywords:** Fetal weight, birth weight, ultrasonography

## Abstract

**Objective:**

To compare the accuracy of clinical and ultrasonographic (USG) estimation of fetal weight in non-complicated, term pregnancies.

**Materials and Methods:**

Two hundred term pregnant women were included in the study. We used three formulae for the estimation of fetal weight at term; the Hadlock formula for the USG method, and two different formulas for clinical methods, maternal symphysis-fundal height and abdominal circumference at the level of umbilicus. Accuracy was determined by mean percentage error, mean absolute percentage error and proportion of estimates within 10% of actual birth weight (birth weight ±10%). Patients were divided into two groups according to actual birth weight, the normal birth weight group (2500-3999 g) and high birth weight group (≥4000 g).

**Results:**

All three methods statistically overestimated birth weight for the high and normal birth weight groups (p<0.001, p=1.000, p=0.233) (p=0.037, p<0.001, and p<0.001). For both groups, the mean absolute percentage errors of USG were smaller than for the other two clinical methods and the number of estimates were within 10% of actual birth weight for USG was greater than for the clinical methods; the differences were statistically significant (p<0.001). No statistically significant difference of accuracy was observed for all three methods for the high birth weight group (p=0.365, p=0.768, and p=0.540). However, USG systematically underestimated birth weight in this group.

**Conclusion:**

For estimation of fetal birth weight in term pregnancies, ultrasonography is better than clinical methods. In the suspicion of macrosomia, it must be remembered that no method is better than any other. In addition, if ultrasonography is used, careful management is recommended because ultrasonography overestimates in this group.

## PRECIS:

We evaluated the effectiveness of ultrasound and clinical examination methods for fetal weight estimation.

## INTRODUCTION

Over the last two or three decades, fetal weight estimation has almost become a routine antepartum test in the management of high-risk pregnancies and birth. The two methods used in modern obstetrics practice are the clinical evaluation of fetus weight and ultrasound. Through the late 1960s, external clinical examination became the most frequently and widely used method for the determination of fetus weight and dimensions. Studies have shown that 80-85% of clinical estimations were between ±500 grams of the actual birth weight (ABW) and 69% were between ±10%^(1,2)^. However, the use of clinical methods has decreased with the increased use of ultrasonographic (USG) for fetal weight estimation in recent years. Studies have shown that the mean absolute percentage failure rate of USG estimations generally range between 6% and 12%, and the estimation rate of actual birth weight, which was ±10%, was between 40% and 80%^(3,4,5,6)^.

Various researchers have suggested that palpation of the uterus is insufficient for the estimation of fetal weight^(7,8)^. Ultrasound, as a technical, objective, and repeatable method, is generally perceived as superior to clinical estimation. Studies in the literature that compared the two methods found different results^(9,10,11)^. These studies often involved estimations through evaluation of uterus size externally with use of a physician’s hands. This method is the oldest and most popular method and there have been doubts about its use because it is not objective. Therefore, it would be beneficial to use a simple, easy, cheap, but standard and objective clinical method as an alternative to USG, which is expensive and not always easy to access, especially in countries with limited financial resources for health. There are objective and standard clinical methods; however, the number of studies that evaluate the effectiveness of these methods or compare them with USG, is quite low.

The purpose of this study was to compare the accuracy of clinical and USG estimation of fetal weight in non-complicated term pregnancies.

## MATERIALS AND METHODS

This prospective single-center study was approved by the local ethics committee of Etlik Zübeyde Hanım Women’s Health Teaching and Research Hospital and informed consent was obtained from all the patients. Two hundred women with term pregnancies, who were consecutively admitted to our department between November 10^th^ 2008, and August 29^th^ 2009, were included in the study. All pregnancies were at 37 gestational weeks with normal amniotic fluid index (50-200 mm), vertex presentations and labor had not yet begun. In a number of patienst, a vaginal examination during labor concluded with a maximum 3 cm dilatation and 60% effacement. The lowest part of the fetal head was over the interspinal line and the engagement had not yet begun. Gestational week was identified through counting the days according to the last menstrual period or a USG measurement performed before the 22^nd^ week of pregnancy. Exclusion criteria were polyhydramnios, preterm labor, multiple pregnancy, intrauterine growth retardation, early membrane rupture, abnormal presentation, antepartum hemorrhage, eclampsia, preeclampsia, gestational diabetes mellitus (GDM), known congenital fetal anomaly, systemic disease in the mother, and placental anomalies.

Patients that fulfilled the study criteria were registered on the monitoring chart including their name and surname, age, height, weight, gravida, parity and pregnancy age. Next, a clinical examination was made when they were lying on their back with their hands on both sides, legs stretched, and with an empty bladder. First, the distance between the symphysis pubis mid point of the top corner and the uterus fundus top point was measured on the naked abdomen of the mother using a non-flexible, curved tape measure (SPFU). In addition, abdominal circumferences of the mother at the umbilicus level (AP) were measured in centimeters. Ultrasound predicted fetus weight was then measured in grams and recorded. All USG measurements were performed using a Logic P 5 USG device, which had a 3200 MHz trans-abdominal probe; a three-parameter Hadlock formula [biparietal diameter (BPD), femur length (FL), and abdominal circumference (AC)] was used. All sonography and clinical measurements were performed on the same day by the same researcher, who was in his fifth year as an assistant.

For the BPD measurement, the greatest distance between the outside corner of the front parietal bone and the inner corner of the back parietal bone was measured at a right angle using electronic indicators on the frozen image, where falx cerebri echo was observed at the mid-line, the thalamus were monitored symmetrically on both sides; the cavum septum pellucidum was ⅓ of the distance from the frontal occipital. On the same platform, occipitofrontal diameter (OFD) was measured from the outside corner of the frontal bone to the outside corner of the occipital bone, parallel to the falx cerebri with a right angle to the BPD. Cephalic index (BPD/OFDx100) was calculated in order to exclude dolichocephaly and brachiocephalic. The cephalic index value was included in the study and was between 0.77 and 0.83. Where all long axes of the femur were monitored, the distance between the two ends of the diaphysis was measured for FL, without including the femoral head and distal epiphysis. With respect to the AC measurement, first, using the fetal spine or fetal aorta, the direction of the longitudinal axis of the fetal body was identified. The fetal umbilical vein was obtained, along with a circular and symmetric abdominal image at a right angle to these structures at the level of the stomach. On the platform, where the umbilical vein was monitored at the front ⅓ it gave the portal branch, the stomach was monitored without monitoring the heart and bladder, and a chamber surrounding the skin echo from the outside corner to the other outside corner was created and measurements were performed. Following the recording of the three biometric data, fetal weight was automatically measured and recorded using the USG device. The mother was monitored in the delivery room.

Two formulas were used for clinical estimations. In the first formula, SPFU and AP were multiplied in centimeters and TFW was calculated in grams.

**Formula 1:** TFW (g)=SPFU (cm) x AP (cm)

The Jonson formula was used as the second formula.

**Formula 2:** TFW (g)=(SPFU (cm) - n) x 155 (n=12 was used because engagement was not experienced in all cases). Where the weight of the mother was equal to more than 91 kg, 1 cm was reduced from the fundus height.

Fetal weight estimations obtained through the three different methods were registered in the monitoring chart. Finally, a nurse measured actual birth weight in grams using an electronic scale within the first 10 minutes after birth; actual weight was recorded along with the time between examinations and birth.

The patients in the study were divided into two groups according to the postpartum measured actual fetal birth weight; group 1 with actual birth weight of 2500-3999 g and group 2 with actual birth weight t equal to or more than 4000 g. In order to determine the performance of USG: formula 1 and formula 2 were used to predict intrauterine fetal weight at the prenatal stage; and mean failure percentage (MFP) [(predicted birth weight-actual birth weight) x 100/actual birth weight]; mean absolute failure percentage (MAFP) (absolute value (predicted birth weight -actual birth weight) x 100/actual birth weight) values; and the percentage of estimations that could predict actual birth weight within ±5%, ±5.1-10.0%, ±10.1-20.0%, and more than 20.0% of failure were calculated separately for each method in all three groups. In all groups, the amount of failure in estimations made using the Bland-Altman method (bias) and 95% confidence intervals of the fitness level were calculated. Furthermore, estimation rates belonging to the standard deviation value of 1 and standard deviation value of ±375 g were calculated in all groups for formula 1 and formula 2.

## RESULTS

Two hundred women whose pregnancies were at the 37th gestational week with normal amniotic fluid index (50-200 mm), vertex presentations, and labor had not yet begun, were included in the study. Demographic characteristics of the women are shown in Table 1.

Birth weight was less than 4000 grams in 88% (n=176) of the women and more than 4000 grams in 12% (n=24). In the group where actual birth weight was less than 4000 g, and fetal weights were estimated with USG, formula 1 and formula 2 were statistically significantly higher (p=0.037, p<0.001, and p<0.001), respectively. Birth weights estimated with formula 1 and formula 2 were statistically significantly higher than birth weights estimated with USG (p<0.001). In addition, birth weights estimated with formula 2 were statistically significantly higher than birth weights estimated with formula 1 (p<0.001) (Table 2).

**Figure 1:**
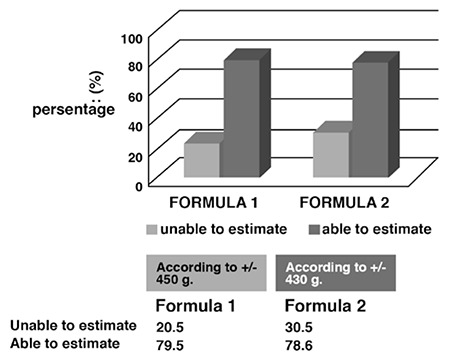
Estimation rates for formula 1 and formula 2 for 1 standard deviation level

In the group where the actual birth weight was 4000 g and higher, birth weight estimated with USG was statistically significant and a mean of 201 g less than the actual birth weight (p<0.001). Birth weight estimated with formula 1 was a mean 39 g less than actual birth weight and was not found to be statistically significant (p=1.000). Birth weight estimated with formula 2 was a mean 48 g less than actual birth weight and again was not found to be statistically significant (Figure 1). The difference between USG and formula 1 was not found to be statistically significant (p=0.233), but birth weight estimated with formula 2 was 249 g higher than with USG, which was statistically significant (p=0.008). In addition, the mean difference estimated with formula 1 was 88 g higher than with formula 2; this was not statistically significant (p=0.642) (Table 3).

## CONCLUSION

Although some researchers found sonographic estimations better than clinical estimations,^(9,10)^ others reported that the success of both methods was similar or that the clinical method was better^(11,12)^. The differences in the conclusions might have been influenced by non-homogenous samples: term and preterm fetuses were evaluated together, maternal demographic characteristics were different, small sample sizes, different sonographic and clinical methods used, and variations in definitions and diversity of statistical analyses performed. It is important to ensure standardization in such studies because of the redundancy of reasons that cause differences. In order to do this, in our study we included pregnant women who were in the 37th week or later stage of pregnancy, whose actual birth weight is 2500 g and higher, who are singular, in vertex presentation, with Anxiety sensitivity index (ASI) within normal limits, no fetal macroscopic anomalies detected before and after birth, no known maternal systemic disease and complications, and who were not yet in labor or whose labor had just started. Principally, we aimed to assess ultrasound and clinical examination of fetal weight estimation in term pregnancies under normal conditions.

In studies of fetal weight estimation, generally two statistical parameters have been used in the evaluation of effectiveness; Mean absolute percentage error (MAPE) (with Mean error percentage) and estimation rates were within ±5-10% of actual birth weight. In the literature, it was observed that the MAPE given for the success of sonographic estimations was between 6% and 12% and the estimation rate within ±10% was between 40% and 80%^(9,10)^. When we looked at the success rates of clinical estimations, 80-85% of estimations were within ±500 g of the actual birth weight and 55-72% of estimations were within ±10% of the actual birth weight; the MAPE varied between 7.5% and 19.8% in term pregnancies^(1,2)^.

In our study, it was observed that all three methods estimated birth weight systematically higher in the group in which actual birth weight was less than 4000 g. According to the Bland-Altman method, the differences among groups were found to be statistically significant. The order of most successful estimations was USG >formula 1 >formula 2. In the group in which birth weight was 4000 g and higher, both MAPE values and rates of estimation within ±5% and ±10% ranges were found to be similar. As in the group with normal birth weight, USG was most successful, formula 1 was on moderately successful, and formula 2 was least successful in terms of estimation within acceptable limits for all participants.

In the group in which birth weight was higher than 4000 g, the MAPE values of all three methods were lower and estimation rates within ±10% were higher compared with the other two groups. However, it was observed that MAPE values (p=0.452 and p=0.369), and estimation rates of actual birth weight within ±10% (p=0.432 and p=0.632) were similar for USG and formula 1 in both <4000 g and ≥4000 g groups; there was no statistically significant difference between them. However, although the MAPE values were found to be lower for formula 2 in the ≥4000 g group compared with the <4000 g group (p=0.025), the estimation rate within ±10% was found to be significantly higher (p=0.046). Consequently, it can be said that formula 2 was successful with large fetuses.

With few exceptions, standard and objective methods have not been used for clinical estimation in previous studies; estimations were made by examining the uterus with external palpation and with Leopold maneuvers. In this study, we used 2 more objective and standard clinical examination methods. Until the early 1990’s, it was generally accepted that USG was superior to clinical estimation in fetal weight estimation without any scientific grounds. Clinical methods were largely neglected in line with the popularization of USG devices. Following the studies of Chauhan and many other researchers in which the authors reported that clinical estimations were as successful as USG, prejudices on this matter started to change^(13,14,15)^. Sherman et al.^(9)^ compared external examination and USG (Shepard-Hadlock) in 1717 fetuses with a mean birth weight of 3334 g, 85% of which were term and had no membrane rupture. The authors observed that USG systematically estimated lower in the 2500-4000 g range and the estimation rate within ±10% was 70.6%. There were no systematic errors in the clinical method whose rate was 75.1%; the authors concluded that the clinical method was superior to USG for normal-weight fetuses. For 4000 g and higher, they found that estimation rates within ±10% were 58.8% for USG and 61.3% for the clinical method; both methods estimated systematically lower but there was no statistically significant difference between the two methods. The lowest error and highest estimation rates with both methods were obtained in the group of fetuses with normal weight^(15)^. Baum et al.^(16)^ reported that USG was not superior to the clinical and mother’s estimation, and Watson et al.^(11)^ reported that there was no difference between the two methods, even in extreme term weights^(16)^. In two different studies that compared the two methods, error rates were found to be less than 10% and no superiority of one method over the other was reported^(17)^. One of the few studies in which the effectiveness of the clinical method compared to USG in terms of fetal weight estimation was carried out by Shittu et al.^(18)^ in Nigeria in 2004. In their study of 100 pregnant women, which resembles our population, the authors compared Hadlock 2^(19)^ and formula 1 and reported that neither of the two methods was superior the other in the whole sample of 2500-3999 g and 4000 g and higher fetuses. In all three groups, systematically lower estimations were made with USG and higher estimations were made with clinical methods. In Shittu et al.^(18)^ study, the number of fetuses that weighed 4000 g and higher was 17. When we compared the MAPE values and the estimation rates within ±10% for 24 patients in the same group in our study with those found by Shittu et al.,^(18)^ it was observed that better results were obtained in large fetuses with both methods, except in the ±10% ratio for formula 1 (Table 4).

Görgen et al.^(20)^ estimated fetal weight in 574 singular pregnant women who were in the 34th-42nd week of pregnancy, whose actual birth weight was 2050-5550 g, and had variable vertex presentation with Johnson formula (formula 2) by measuring SPFY and reported that 72.3% of their measurements were within ±10% of actual birth weights^(20)^. The estimation rate of Görgen et al.^(20)^ for this difference was 75.6%. In our study, the estimation rate within ±10% for formula 2 was 56%, the estimation rate for ±375 g level was 62%, and for 1 SD value of ±430 g was 78.6%, which were similar to the other two studies.

Physicians will not always encounter pregnant women with standard characteristics as in this study. Therefore, it is necessary to investigate the effectiveness of USG in different pregnant populations. These can be listed as the most frequently encountered preterm labor, fetuses lighter than 2500 g, oligohydramnios, early membrane rupture, preterm premature rupture of membranes, breech presentation, maternal obesity, and GDM.

In our study, labor did not begin in all groups and engagement of fetal head had not occurred in the latent-or early phase. Amniotic membranes were intact, ASI was within normal limits, and all fetuses were in vertex presentation. In a study of 218 fetuses, Çelik^(21)^ reported that sonographic estimations, carrying out the labor in active and latent phase, and presence of membrane rupture had no significant impact on the success of estimation^(22)^. Benacerraf et al.^(3)^ reported that oligohydramnios and polihydramnios did not affect the estimation success following their study of 1301 fetuses, whereas Barnhard et al.^(23)^ suggested that estimations should be made before amniotomy because oligohydramnios significantly reduces the success of sonographic and clinical estimations. Scioscio et al.^(24)^ determined no difference between cephalic and non-cephalic presentations. Blann^(25)^ reported that cervical dilatation and head level were not effective on sonographic and clinical estimation.

In our study, ultrasonographic and clinical measurements were carried out on the same day and all deliveries occurred within seven days of the estimation. Some 90.5% of the deliveries occurred on the same day and no corrections were made for time difference. The weight of fetuses increased by a mean 25-30 g a day and 200-250 g a week during term. In the study of Benacerraf et al.^(3)^ on 1301 pregnant women, intervals varied between 0-7 days as in our study, and 80% of the women gave birth between the 1st and 5th days. The additional time did not affect the results^(3)^. No corrections were made for additional time because all of the births in our study occurred within 7 days and 94% occurred within the first 48 hours.

These results will be valuable and useful in groups with similar characteristics to the patients in this study. However, pregnant women with these characteristics will not be encountered in daily practice. Therefore, studies should be performed that compare USG and objective clinical examination methods, various commonly used ultrasound methods, and include various obstetric populations such as preterm labor, membrane rupture, various labor phases, GDM, non-vertex presentations.

## Figures and Tables

**Table 1:**
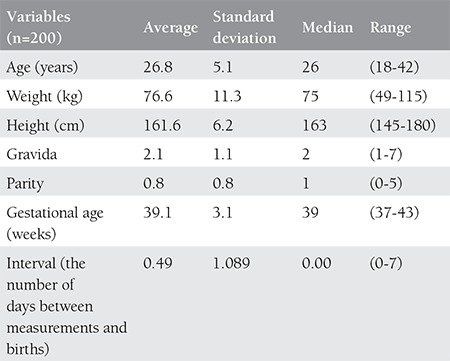
Demographical characteristics of all cases

**Table 2:**
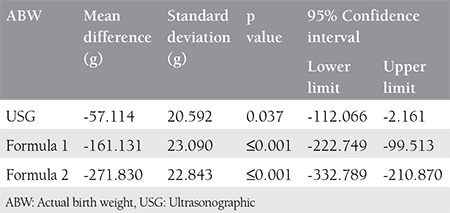
Comparison of actual birth weight (ABW) with ultrasonographic, formula 1 and formula 2 using Bonferroni corrected multiple comparison test in patients with ABW <4000 g

**Table 3:**
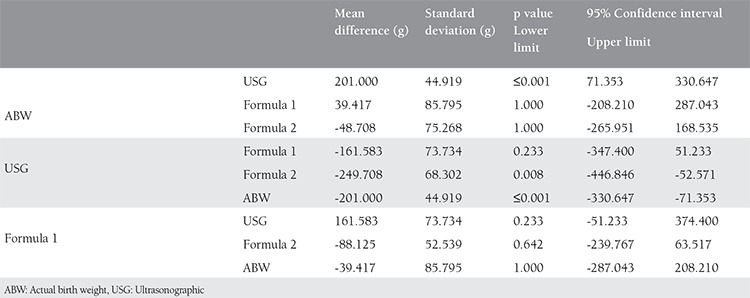
Comparison of actual birth weight (ABW), ultrasonographic, formula 1 and formula 2 byusing Bonferroni corrected multiple comparison test in patients with ABW ntsents

**Table 4:**
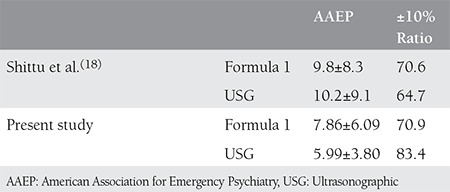
Comparison of Shittu et al.(18) and present study for formula1
